# Past trends in obesity-attributable mortality in eight European countries: an application of age–period–cohort analysis

**DOI:** 10.1007/s00038-018-1126-2

**Published:** 2018-06-04

**Authors:** Nikoletta Vidra, Maarten J. Bijlsma, Sergi Trias-Llimós, Fanny Janssen

**Affiliations:** 1 Population Research Centre, Faculty of Spatial Sciences, University of Groningen, PO Box 800, 9700 AV Groningen, The Netherlands; 2 Max Planck Institute for Demographic Research, Rostock, Germany; 3 Netherlands Interdisciplinary Demographic Institute, The Hague, The Netherlands

**Keywords:** Obesity, Mortality, Birth cohort, APC analysis, Europe

## Abstract

**Objectives:**

To assess age, period, and birth cohort effects and patterns of obesity-attributable mortality in Czech Republic, Finland, France, Germany, Hungary, Italy, Poland, and the UK (UK).

**Methods:**

We obtained obesity prevalence and all-cause mortality data by age (20–79), sex and country for 1990–2012. We applied Clayton and Schifflers’ age–period–cohort approach to obesity-attributable mortality rates (OAMRs).

**Results:**

Between 1990 and 2012, obesity prevalence increased and age-standardised OAMRs declined, although not uniformly. The nonlinear birth cohort effects contributed significantly (*p* < 0.01) to obesity-attributable mortality trends in all populations, except in Czech Republic, Finland, and among German women, and Polish men. Their contribution was greater than 25% in UK and among French women, and larger than that of the nonlinear period effects. In the UK, mortality rate ratios (MRRs) increased among the cohorts born after 1950. In other populations with significant birth cohort effects, MRRs increased among the 1935–1960 cohorts and decreased thereafter.

**Conclusions:**

Given its potential effects on obesity-attributable mortality, the cohort dimension should not be ignored and calls for interventions early in life next to actions targeting broader societal changes.

**Electronic supplementary material:**

The online version of this article (10.1007/s00038-018-1126-2) contains supplementary material, which is available to authorized users.

## Introduction

Obesity has increased dramatically in recent decades and is now considered a global epidemic (Finucane et al. 2011). While the United States (USA) still has the highest obesity prevalence (OECD 2014) (36.5% in 2011–2014), Europe ranks second (Eurostat 2017), with national prevalence levels ranging from 10 to 30% (Eurostat 2017). Obesity has major consequences for individual and for population health, as it increases the risk of a range of diseases (Field et al. 2001) and consequently the risk of dying (Global BMI Mortality Collaboration 2016). In order to set effective health policy priorities, it is essential to study the obesity epidemic and gain an understanding of how it evolved over time (Finucane et al. 2011).

Previous studies that examined time trends in obesity prevalence have provided valuable insight into how the epidemic evolved, the differences that exist across countries and between men and women, and the underlying factors (Finucane et al. 2011). Previous studies that focused on estimating the health burden of obesity by assessing obesity-attributable mortality were mostly conducted in the USA (Allison et al. 1999; Flegal et al. 2005; Mehta and Chang 2009) and to a lesser extent in Europe (Banegas et al. 2003; Kelly et al. 2009) and did not assess trends over time. To the best of our knowledge, only one previous study has examined the long-term trends in the share of mortality due to obesity in Canada indicating that this share increased over time (Katzmarzyk and Ardern 2004).

Few of the aforementioned studies took into account the multiple dimensions of the obesity epidemic: namely, age, period, and birth cohort (Reither et al. 2009). It is well known that age affects obesity, as declining levels of physical activity and physiological changes occur with increasing age (Reither et al. 2009). In addition, broad societal changes, such as the increase of sedentary lifestyles and the consumption of high-calorie foods, have contributed to increases in obesity over calendar time (period effects) (Masters et al. 2013; Reither et al. 2011). However, differences in the obesity prevalence levels among people born in different years (i.e., (birth) cohort effects) exist as well (Reither et al. 2011).

Birth cohort membership is important because it could indicate the degree to which individuals are receptive to societal and social changes (Hellevik 2002; Reither et al. 2009). Compared to older birth cohorts, younger birth cohorts are more likely to have a high-calorie diet and a sedentary lifestyle, largely because they are more receptive to using modern technologies, products, and media (Reither et al. 2009). Birth cohort effects may also reflect long-lasting effects of exposures earlier in life (Janssen and Kunst 2005). A growing body of evidence indeed shows that excess fat in adolescence or early adulthood and weight gain over the life course have long-term implications for metabolic, cardiovascular, and mortality risk (Yu 2012). Because of these long-lasting effects, birth cohort dynamics are key to understanding the future of health and longevity (Masters et al. 2013).

The few previous studies that have applied age–period–cohort (APC) analyses to obesity prevalence found that age, period, and cohort effects were all important (Diouf et al. 2010; Jiang et al. 2013; Masters et al. 2013). While there are no APC analyses of obesity-attributable mortality, Yu applied APC analysis to mortality differentials associated with body mass (Yu 2012), and Masters estimated obesity-attributable mortality by birth cohort and found more deaths among younger than older birth cohorts (Masters et al. 2013). However, neither study focused on Europe.

Our study is the first to assess age, period, and birth cohort effects and patterns in past obesity-attributable mortality trends in Europe.

## Methods

### Design

We estimated obesity-attributable mortality trends from 1990 to 2012 for the adult populations of eight European countries: the Czech Republic, Germany, Finland, France, Hungary, Italy, Poland, and the UK.

To warrant the data quality, we included only European countries for which the trends in obesity prevalence, which we obtained from the Global Burden of Disease (GBD) study (Ng et al. 2014), revealed similar patterns as those based on data from the Organisation for Economic Co-operation and Development (OECD 2016). Also, we ensured that the included countries comprise the four different European regions and differ in terms of the levels, patterns, and trends in obesity prevalence. To ensure that the obesity prevalence data and the all-cause mortality data were closely aligned, we restricted our analysis to the period 1990–2012. We focused on the adult population aged 20–79, because the prevalence data had 80+ as the final open-ended age category with unknown upper. The combination of ages 20–79 and calendar years 1990–2012 resulted in the inclusion of the birth cohorts from 1911 to 1992 (cohort = period − age).

### Data

Estimated obesity prevalence data (BMI ≥ 30 kg/m^2^) (WHO 1998) were obtained from the GBD study by country, five-year age groups, sex, and single calendar year for the period 1990–2012 (Ng et al. 2014). These data were generated by using a spatiotemporal regression and a Gaussian process regression, which allow dealing with the available prevalence data from different sources and missing data (Ng et al. 2014).

We obtained all-cause death numbers and exposure population numbers by country, sex, five-year age groups, and year from the Human Mortality Database (2016).

### Analysis

We performed all of our analyses separately for men and women.

#### Obesity-attributable mortality

To calculate the obesity-attributable mortality fractions by country, year, age and sex, that we shall refer to as obesity-attributable mortality fraction (OAMF), we used the Rockhill formula for the population-attributable fraction (PAF) (Rockhill et al. 1998):

$${\text{OAMF}}_{a,s} = \frac{{P_{a,s} \cdot \left( {{\text{RR}} - 1} \right)}}{{1 + P_{a,s} \cdot \left( {{\text{RR}} - 1} \right)}}$$

where $P_{a,s}$ is the obesity prevalence in five-year age group *a* and sex *s* by country and year and RR the relative risk of mortality associated with obesity (Rockhill et al. 1998). We used the RR from a recent meta-analysis based on European studies that adjusted for at least age, sex, and smoking (Flegal et al. 2013). In using an adjusted RR in the Rockhill formula, we basically applied the “partially adjusted method” for estimating PAFs (Flegal et al. 2004). Because the sex differences in the RRs were only marginal, we used the overall RR of 1.27 (Flegal et al. 2013).

Age- and sex-specific obesity-attributable mortality deaths were estimated by multiplying age- and sex-specific OAMF by the corresponding deaths. All-age obesity-attributable mortality fractions were obtained by dividing the sum of age-specific obesity-attributable mortality deaths by the total deaths for each sex.

The obesity-attributable mortality rates (OAMRs) were obtained by dividing the age- and sex-specific obesity-attributable deaths by the corresponding exposure populations. These five-year age group obesity-attributable mortality rates were subsequently turned into single age-specific mortality rates by applying two-dimensional P-splines smoothing (Camarda 2012).

#### Descriptive analysis

The age-specific obesity-attributable mortality rates by birth cohort were depicted in graphs, where we have selected single ages 5 years apart, to improve the visual clarity. The obesity-attributable mortality rates and obesity prevalence levels were directly age-standardised to ensure their comparability across countries, thereby using the sex-specific European population of 2011 as a standard (Eurostat 2016).

#### Age–period–cohort (APC) modelling

We applied age–period–cohort (APC) modelling to obesity-attributable mortality rates. The section below follows largely the APC methodology, as described by Bijlsma et al. (2012).

In modelling the obesity-attributable mortality rates as a function of age, period, and birth cohort, the linear dependency between the three variables (*a* = *p* − c) needs to be taken into account to avoid over-identification. We therefore applied the standard Clayton and Schifflers approach (Clayton and Schifflers 1987a, b). This method, which has been used in previous demographic (Janssen and Kunst 2005) and epidemiological studies (Bijlsma et al. 2012; Dhillon et al. 2011), distinguishes between age effects, the effect of the shared linearity of period and birth cohort (referred to as drift), the nonlinear period effects, and the nonlinear birth cohort effects.

We applied four Poisson regression models to obesity-attributable death numbers for each county and sex combination, setting the natural logarithm of the exposure population as an offset term. The age (A) model is defined by: $\ln \left[ {m_{a} } \right] = \mu + \alpha_{a}$; the age–drift (AD) model is defined by $\ln \left[ {m_{ad} } \right] = \mu + \alpha_{a} + \delta$; the age–period (AP) model is defined by $\ln \left[ {m_{ap} } \right] = \mu + \alpha_{a} + \beta_{p}$; and the age–period–cohort (APC) model is defined by $\ln \left[ {m_{apc} } \right] = \mu + \alpha_{a} + \beta_{p} + \gamma_{c}$. Here, *m* is the obesity-attributable mortality rate, *µ* is the intercept, *α*, *β,* and *γ* represent the age, period, and birth cohort effects, and *δ* represents the drift (Janssen and Kunst 2005).

We used as constraints two categories of birth cohorts, as this approach allowed us to estimate and visualise the nonlinear birth cohort effects separately from the linear time trend changes. As reference categories, we used age 50, calendar year 2000, and the 1935 and 1970 birth cohorts. Age 50 and calendar year 2000 represent the middle observations of our data (1990–2012; 20–79). The two reference categories for the cohort dimension were chosen so that they do not refer to the extreme birth cohorts with fewer observations, but still lie as much apart as possible so as to separate a long-term linear trend from the nonlinear deviations of that trend (Bijlsma et al. 2012; Trias-Llimos et al. 2017). For comparative purposes, we used the same reference categories for all countries and both sexes in our analysis.

To determine the statistical contribution of birth cohort to observed trends and levels, we compared the goodness of fit of age (A), age–drift (AD), age–period (AP), and age–period–cohort (APC) for the different models. Using the deviance statistic, a measure of goodness of fit, we performed both likelihood ratio tests of the A, AD, AP, and APC models to the data, and likelihood ratio tests for model reduction. Due to our interest in birth cohorts, the primary comparison for the model reduction tests was APC with AP, but we also compared AD with A and AP with AD. Reduction in deviance is expressed as percentage reduction in deviance between the age-only model and the APC model. In our graphs, we show the cohorts born between 1920 and 1980, as observations for more extreme cohorts were less complete. All of the data analyses were performed using R 3.2.5 in R studio 0.99.451.

## Results

**Fig. 1:**
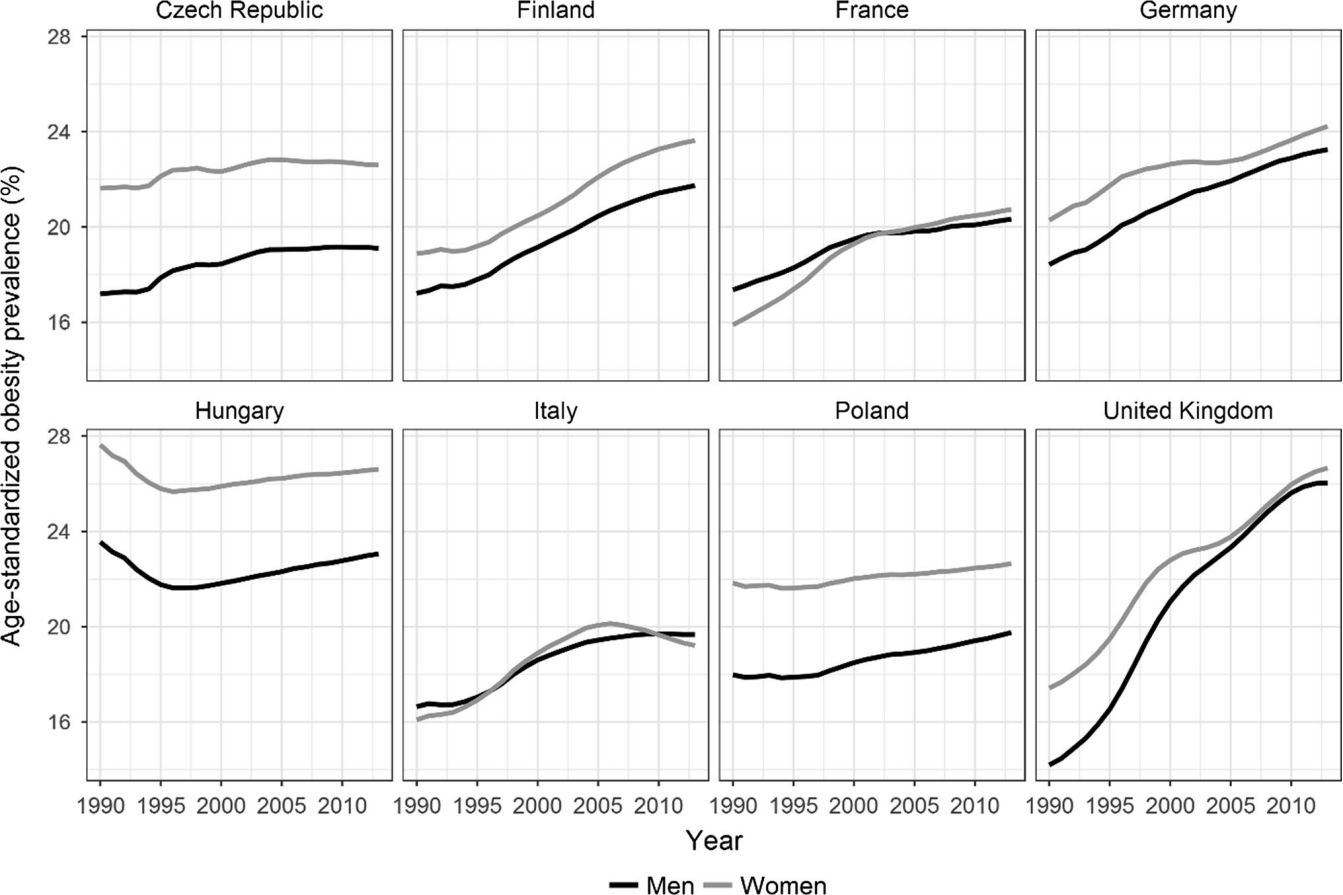
Age-standardised obesity prevalence trends by sex in populations aged 20–79 years in eight European countries, 1990–2012

Over the calendar years 1990–2012, age-standardised obesity prevalence increased among the adult populations in all eight countries, albeit not to the same extent (Fig. 1). The biggest increases were in the UK, followed by Finland and Germany. In the Czech Republic, France, Poland, Italy, Hungary, and Germany (women only), rising obesity periods were accompanied by periods in which a levelling off occurred. Age-standardised obesity prevalence was generally higher among women than men. In 2012, obesity prevalence was highest in the UK (26%) and was lowest (less than 20%) in Italy and among men in the Czech Republic and Poland.

The trends in the sex-specific obesity-attributable mortality fraction (OAMF) look similar to the trends in age-standardised obesity prevalence except for Hungarian and Polish women (see Online resource Fig. S1). In 2012, the share of all-cause mortality due to obesity ranged from 5.43% (France) to 7.07% (UK) among men, and from 4.85% (France) to 8.21% (Germany) among women. Between 1990 and 2012, the OAMF increased in all of the countries except in Hungary and among Polish women, albeit not to the same extent.

**Fig. 2:**
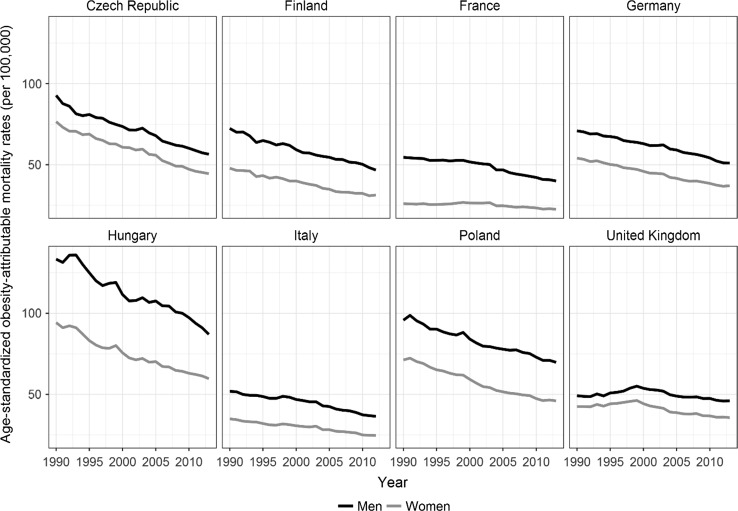
Age-standardised obesity-attributable mortality rates (OAMRs) by sex in populations aged 20–79 years in eight European countries, 1990–2012

Over time, the age-standardised obesity-attributable mortality rates (OAMRs) declined in all of the populations studied, although not uniformly (Fig. 2). The pace of the decline was relatively fast in Hungary, the Czech Republic, and Poland, but was relatively moderate throughout the period in Finland, Germany, and Italy. Among French women and British men and women, the OAMRs increased prior to 2000. Over the same period, the age-standardised all-cause mortality rates decreased faster than the age-standardised OAMRs (see Online resource Fig. S2).

**Fig. 3:**
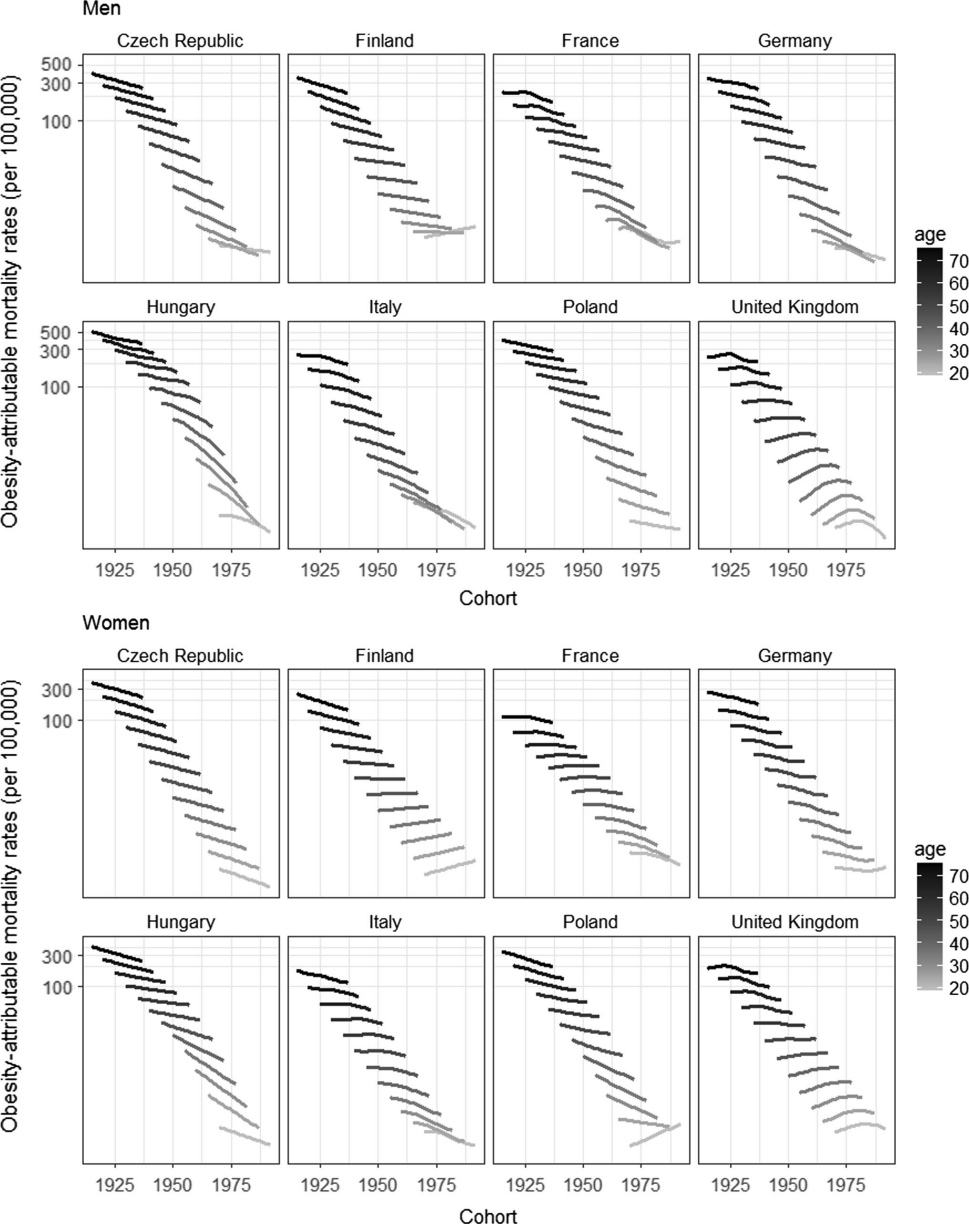
Age-specific obesity-attributable mortality rates (OAMRs) by birth cohort (1911–1992) (log scale) and sex, in populations aged 20–79 years in eight European countries, 1990–2012

For the majority of the countries, the age-specific obesity-attributable mortality rates showed an overall decline across birth cohorts. However, for the UK especially we observed increasing obesity-attributable mortality rates across younger birth cohorts (Fig. 3).

Our modelling of the obesity-attributable mortality trends showed that, in addition to the age effects, both the nonlinear period effects and the nonlinear cohort effects proved to be significant (see Table 1). The contribution of the nonlinear birth cohort effects to the obesity-attributable mortality trends was statistically significant (*p* values < 0.01) among French, Hungarian, Italian, and British men and women and among German men and Polish women (Table 1). For all sex-specific populations, the APC models provided a good fit to the data (*p* values > 0.999) (see Online Resource Table S1).

**Table 1:** Model reduction (log-likelihood ratio) test of the age–drift, age–period, and age–period–cohort models by sex in populations aged 20–79 years in eight European countries, 1990–2012

Log-likelihood ratio test of model reductions in deviance comparing AD to A, AP to AD, and APC to AP (*p* value)								
	Czech Republic	Finland	France	Germany	Hungary	Italy	Poland	UK
Men								
Age drift (AD)	< 0.001**	< 0.001**	< 0.001**	< 0.001**	< 0.001**	< 0.001**	< 0.001**	< 0.001**
Age period (AP)	> 0.999	> 0.999	< 0.001**	< 0.001**	0.096	< 0.001**	> 0.999	< 0.001**
Age period cohort (APC)	> 0.999	> 0.999	< 0.001**	< 0.001**	< 0.001**	< 0.001**	0.665	< 0.001**
Women								
Age drift (AD)	< 0.001**	< 0.001**	< 0.001**	< 0.001**	< 0.001**	< 0.001**	< 0.001**	< 0.001**
Age period (AP)	> 0.999	> 0.999	< 0.001**	< 0.814	> 0.999	< 0.001**	0.944	< 0.001**
Age period cohort (APC)	> 0.999	0.923	< 0.01**	0.351	< 0.01**	< 0.001**	< 0.001**	< 0.001**

Log-likelihood ratio test of model reductions in deviance comparing AD to A, AP to AD, and APC to AP

Statistical significance at *p* value < 0.05, **p value < 0.01

**Fig. 4:**
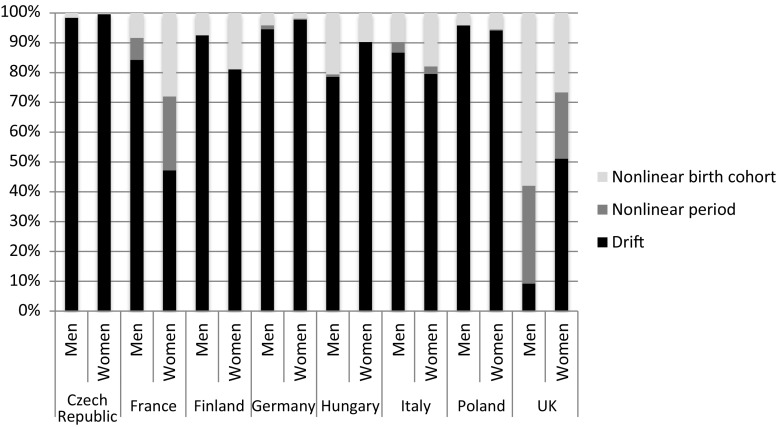
Contribution of the drift, the nonlinear period effect and the nonlinear cohort effect to the deviance reduction between the age model and the age–period–cohort model applied to obesity-attributable mortality, by sex in populations aged 20–79 years in eight European countries, 1990–2012

The contribution of the nonlinear birth cohort effect to the model fit ranged from 1.60% (Czech Republic) to 57.9% (UK) in men and in women from 0.25% (Czech Republic) to 28% (France). The largest contribution of the nonlinear birth cohort effects was among British men (57.9%), while the nonlinear birth cohort contributed more than 25% to the deviance reduction among French and British women. In all of the countries and among both men and women, the contribution of the nonlinear birth cohort effects was larger than the contribution of the nonlinear period effects. The drift made the largest contribution to the deviance reduction, exceeding 75% in all countries and among both men and women, except in the UK and among French women (Fig. 4).

**Fig. 5:**
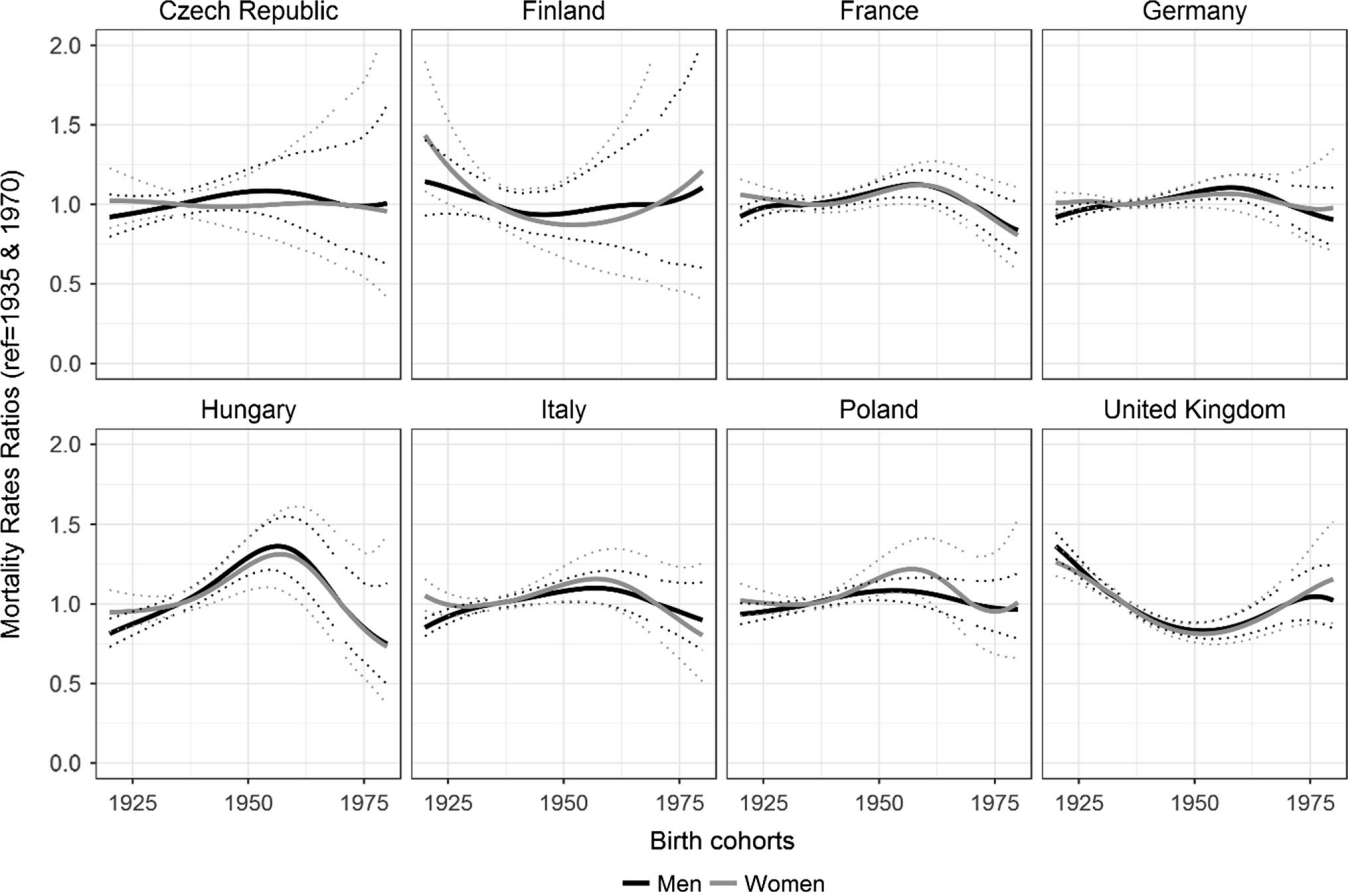
Nonlinear birth cohort patterns by sex in populations aged 20–79 years in eight European countries, 1990–2012

In the UK, obesity-attributable mortality rate ratios started to increase among the cohorts born after 1950 (Fig. 5). This trend was followed by a slight decline among British men born after 1975. In the remaining populations with significant cohort effects (German men; Polish women; and French, Hungarian, and Italian men and women), mortality rate ratios increased among the cohorts born between 1935 and 1960 and decreased among the cohorts born after 1960.

## Discussion

### Summary of results

Between 1990 and 2012, obesity prevalence increased and OAMRs declined in the eight European countries studied, albeit with notable differences across countries, between men and women, and over time. Throughout, the APC models provided a good data fit (*p* > 0.999). Nonlinear birth cohort effects contributed significantly (*p* < 0.01) to the obesity-attributable mortality trends in all of the countries studied, except in the Czech Republic, Finland, Germany (women), and Poland (men). The contribution of these effects was greater than 25% in the UK and among French women, and was larger than the nonlinear period effects. However, drift, the linear component of the period and the cohort, was the largest contributor, except among British men. In general, obesity-attributable mortality rate ratios decreased among the subsequent birth cohorts, except among the cohorts born after 1950 in the UK and among the cohorts of German men; Polish women; and French, Hungarian, and Italian men and women born between 1935 and 1960.

### Evaluation of data and methods

In estimating the share of mortality due to obesity (OAMF), we were hampered by data limitations regarding prevalence and RRs. As obesity prevalence trends were different for different sources, we restricted our analysis to countries for which the obesity prevalence trends from the GBD (Ng et al. 2014) were similar to those by the OECD (OECD 2016). Secondly, and in line with previous studies, we applied adjusted RRs to the Rockhill formula, originally developed for the use of non-adjusted RRs (Flegal et al. 2004), as unadjusted RR were not readily available. Thirdly, the method we used was driven by the availability of data. Although other methods to estimate PAF could have affected the obesity-attributable mortality levels, they would not have affected the trends. Fourthly, because RRs of dying from obesity are not available by country and year, we applied one time-constant European-specific RR (Flegal et al. 2013). A sensitivity analysis where we applied the only available information on declines in RR from the USA (Mehta et al. 2014) revealed that this primarily affected drift and period trends and had no influence on the observed nonlinear cohort trends.

Smoking is importantly affecting overall mortality levels and trends (Thun et al. 2012) and could therefore bias our estimate of obesity-attributable mortality which we obtain by multiplying the obesity-attributable mortality fraction with all-cause mortality. A sensitivity analysis in which we applied our obesity-attributable mortality fractions to non-smoking-related mortality (Janssen et al. 2013) resulted, however, in almost identical cohort patterns.

To differentiate between the age, period, and cohort effects, we used the often-applied Clayton and Schifflers approach (Clayton and Schifflers 1987a, b). By separately modelling the shared linearity of the period and birth cohort effects (referred to as drift), this approach enabled us to estimate the nonlinear period effects and the nonlinear birth cohort effects. The resulting contributions are, however, underestimates of the complete period and cohort effects, which comprise both the nonlinear effects and the linear effects which are embedded in the drift. Especially when drift is large—as it is in our study—the underestimation can be substantial.

The choice of constraints within the APC analysis indeed has the potential to affect the patterns of age, period, and cohort, but not the relative importance of their nonlinear contributions (Fig. 4). A sensitivity analysis, in which we applied two alternative sets of cohort constraints (see Online resource Figs. S3, S4), revealed that our estimated cohort patterns proved in general robust, albeit less so for Hungary and Finland. Therefore, our results and main conclusion were not affected by the choice of constraints.

### Explanation of the results

The decline in the age-standardised OAMRs that we observed might look counterintuitive at first, given the general increase we found in the age-standardised obesity prevalence and in the OAMFs. The more rapid decline of age-standardised all-cause mortality rates compared to the decline in obesity-attributable mortality rates indicates that while obesity indeed had an impact on mortality trends, the observed increase in the OAMFs could not compensate for the greater overall decline in the all-cause mortality rates. For the USA, similar declines in obesity-attributable mortality were observed (Flegal et al. 2005; Mehta and Chang 2009).

Our results show that the cohort dimension is important. Especially for the UK, we were able to demonstrate that nonlinear birth cohort effects made a large contribution to obesity-attributable mortality trends, especially among men (58%) but also among women (27%). The strong cohort effect for the UK can be linked to the finding that more recent UK birth cohorts developed greater probabilities of overweight or obesity at younger ages (Johnson et al. 2015). The observed cohort effect also seems to be another indicator of the further progression of the obesity epidemic in the UK compared to other European countries, next to the higher obesity levels and the sharper increase in obesity prevalence over time (Lifestyles Statistics Team 2014). The cohort patterns for the UK show that obesity-attributable mortality has been increasing among the cohorts born after 1950, which was also reflected in the age-specific obesity-attributable mortality rates by cohorts (Fig. 5).

A similar birth cohort pattern in obesity-attributable mortality rates was observed in the USA (Masters et al. 2013). The consistently lower overweight and obesity prevalence in the UK compared to the USA, combined with a more rapid recent increase (Public Health England 2016), illustrate that the UK is still in the upward dynamic of the obesity epidemic, but that the USA is further ahead. In both phases of the obesity epidemic the cohort dimension thus is important. The evidence showing that there are strong cohort effects and increasing trends in obesity among the younger birth cohorts in these countries points to the need of policy interventions focusing on early life conditions (Reither et al. 2009).

The cohort patterns for the populations among whom the cohort effects were significant—German men; Polish women; and French, Hungarian, and Italian men and women—reveal that the OAMRs increased among the cohorts born between 1935 and 1960, and decreased among the cohorts born after 1960. This cohort pattern is not in line with the increasing levels of receptivity in younger birth cohorts, brought by societal and social changes (Reither et al. 2009). A more likely explanation for the observed increase is that the cohorts born around the time of the Second World War experienced food restrictions even in utero. Poor gestational nutrition may have led to metabolic adaptations of the foetus, thereby increasing the propensity of these cohorts to become obese in adulthood, particularly in an obesogenic environment (Pico et al. 2012).

### Overall conclusion and implications

Next to age and period effects on obesity-attributable mortality, we also observed cohort effects. The substantial birth cohort effect for the UK, with increases in obesity-attributable mortality for those born after 1950, indicates that the UK is following the trajectory of the USA in the obesity epidemic. Other European countries will likely follow the footsteps of the UK and the USA, unless action is being taken. The presence of a cohort effect, reflecting effects that happen early in the life course with long-lasting outcomes, calls for interventions early in life next to actions targeting societal changes which represent period effects.

The cohort dimension should not be ignored in future studies. It provides an important element in our understanding of complex public health problems such as obesity-attributable mortality. Moreover, it can facilitate targeted actions to birth cohorts at elevated risks and—in line with the ageing of current cohorts—inform future obesity-attributable mortality levels.

## Electronic supplementary material

Below is the link to the electronic supplementary material.

Supplementary material 1 (DOCX 618 kb)
