# Supplementary material for: Past trends in obesity-attributable mortality in eight European countries: an application of age–period–cohort analysis
Source: Int J Public Health. 2018 Jun 4;63(6):683–92. doi: 10.1007/s00038-018-1126-2 (PMC6015618; doi:10.1007/s00038-018-1126-2)

**Past trends in obesity-attributable mortality in eight European countries: an application of age-period-cohort analysis.**

**International Journal of Public Health**

Nikoletta Vidra^1^, Maarten J. Bijlsma^2^, Sergi Trias-Llimós^1^, Fanny Janssen^1,3^

1. Population Research Centre, Faculty of Spatial Sciences, University of Groningen, Groningen, The Netherlands
2. Max Planck Institute for Demographic Research, Rostock, Germany
3. Netherlands Interdisciplinary Demographic Institute, The Hague, The Netherlands

Corresponding author:

Nikoletta Vidra_,_ n.vidra@rug.nl

**Appendix**

**Fig. S1** Trends in obesity-attributable mortality fractions by sex in populations aged 20-79 years in eight European countries, 1990-2012


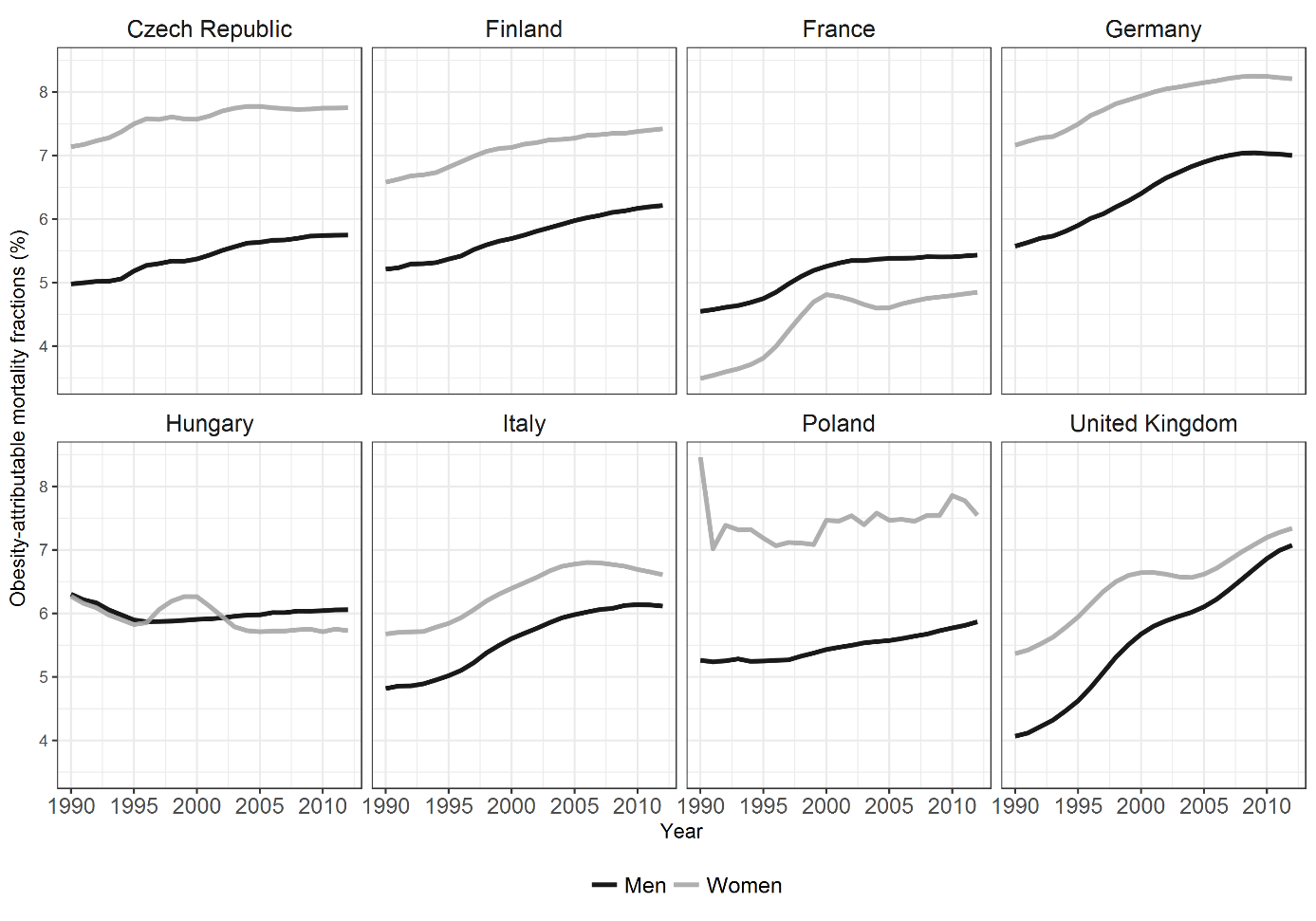


**Fig.S2** Age-standardised all-cause mortality rates by sex in populations aged 20-79 years in eight European countries, 1990-2012

**
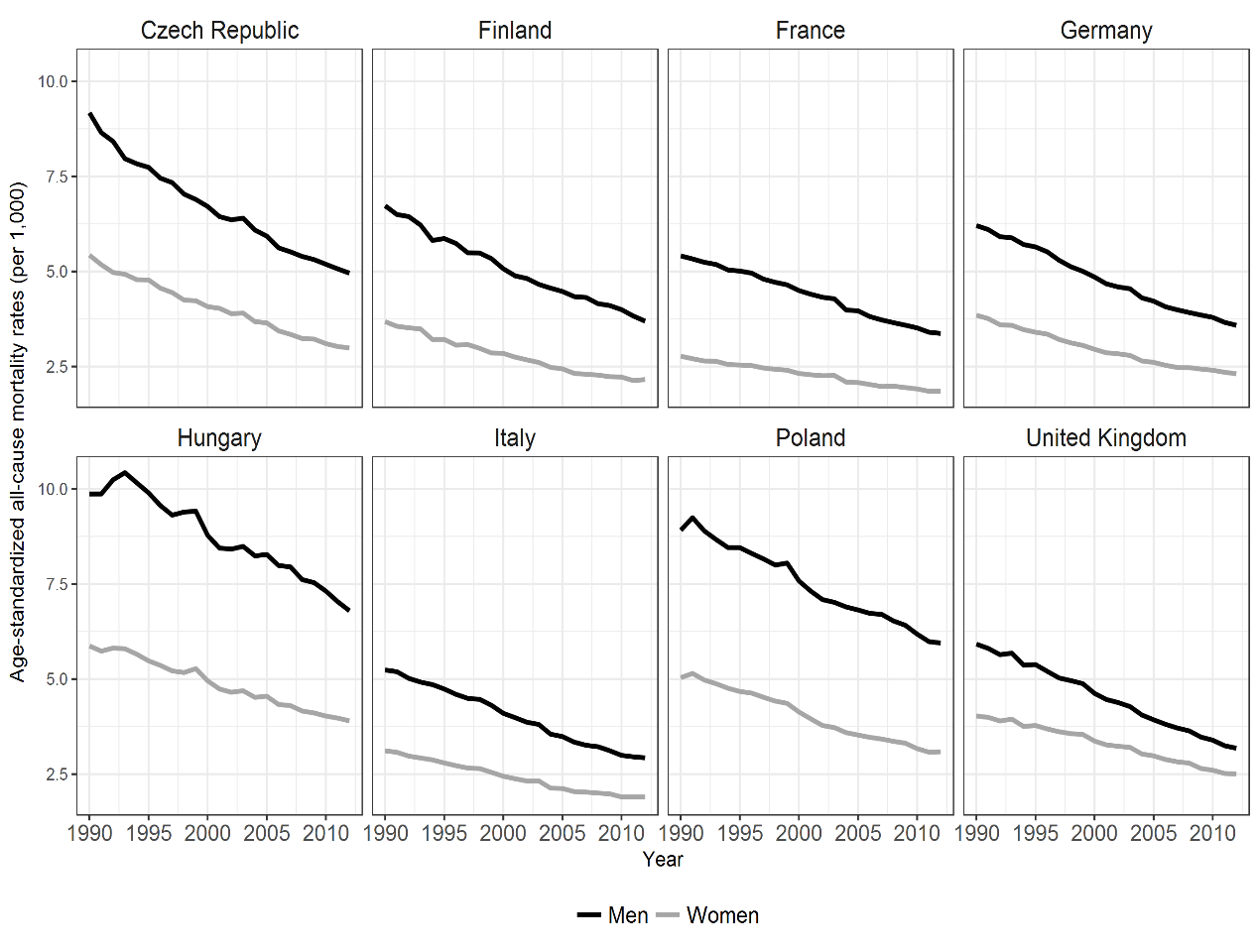
**

**Table S1** Model fit to data (log-likelihood ratio) test comparing all models to the data by sex in populations aged 20-79 years in eight European countries, 1990-2012

| **Log-likelihood ratio test of model fit to data test (p-value)** | | | | | | | | |
| --- | --- | --- | --- | --- | --- | --- | --- | --- |
|  | Czech Republic | Finland | France | Germany | Hungary | Italy | Poland | United Kingdom |
| **Men** | | | | | | | | |
| Age | >0.999 | >0.999 | <0.001** | >0.999 | <0.05* | <0.001** | <0.001** | >0.999 |
| Age drift | >0.999 | >0.999 | >0.999 | >0.999 | >0.999 | >0.999 | >0.999 | >0.999 |
| Age period | >0.999 | >0.999 | >0.999 | >0.999 | >0.999 | >0.999 | >0.999 | >0.999 |
| Age period cohort | >0.999 | >0.999 | >0.999 | >0.999 | >0.999 | >0.999 | >0.999 | >0.999 |
| **Women** | | | | | | | | |
| Age | >0.999 | >0.999 | >0.999 | <0.001** | >0.999 | <0.001** | <0.001** | <0.001** |
| Age drift | >0.999 | >0.999 | >0.999 | >0.999 | >0.999 | >0.999 | >0.999 | >0.999 |
| Age period | >0.999 | >0.999 | >0.999 | >0.999 | >0.999 | >0.999 | >0.999 | >0.999 |
| Age period cohort | >0.999 | >0.999 | >0.999 | >0.999 | >0.999 | >0.999 | >0.999 | >0.999 |

^ᶧ Log-likelihood ratio test comparing model to the data^

^* Statistical significance at p-value< 0.05, **p-value<0.01^

**Sensitivity analysis of the non-linear birth cohort patterns in which two additional sets of cohort constraints were used**

**Fig.S3** Nonlinear birth cohort patterns by sex in populations aged 20-79 years in eight European countries, 1990-2012. Cohort references 1925, 1975, Period and age reference were kept the same (age=50, calendar year=2000).

**
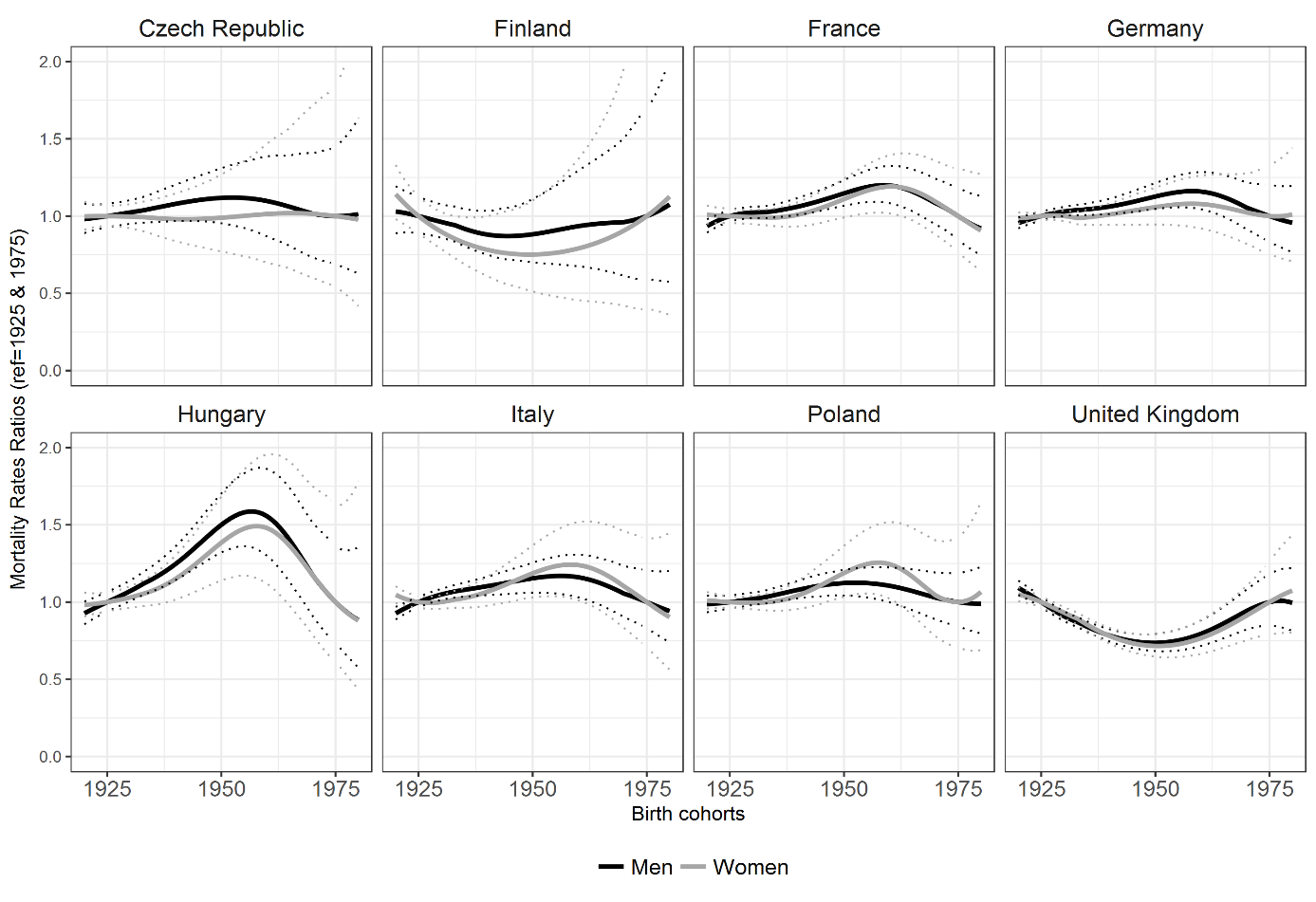
**

**Fig.S4** Nonlinear birth cohort patterns by sex in populations aged 20-79 years in eight European countries, 1990-2012. Cohort references 1940, 1980, Period and age reference were kept the same, age=50, calendar year=2000.


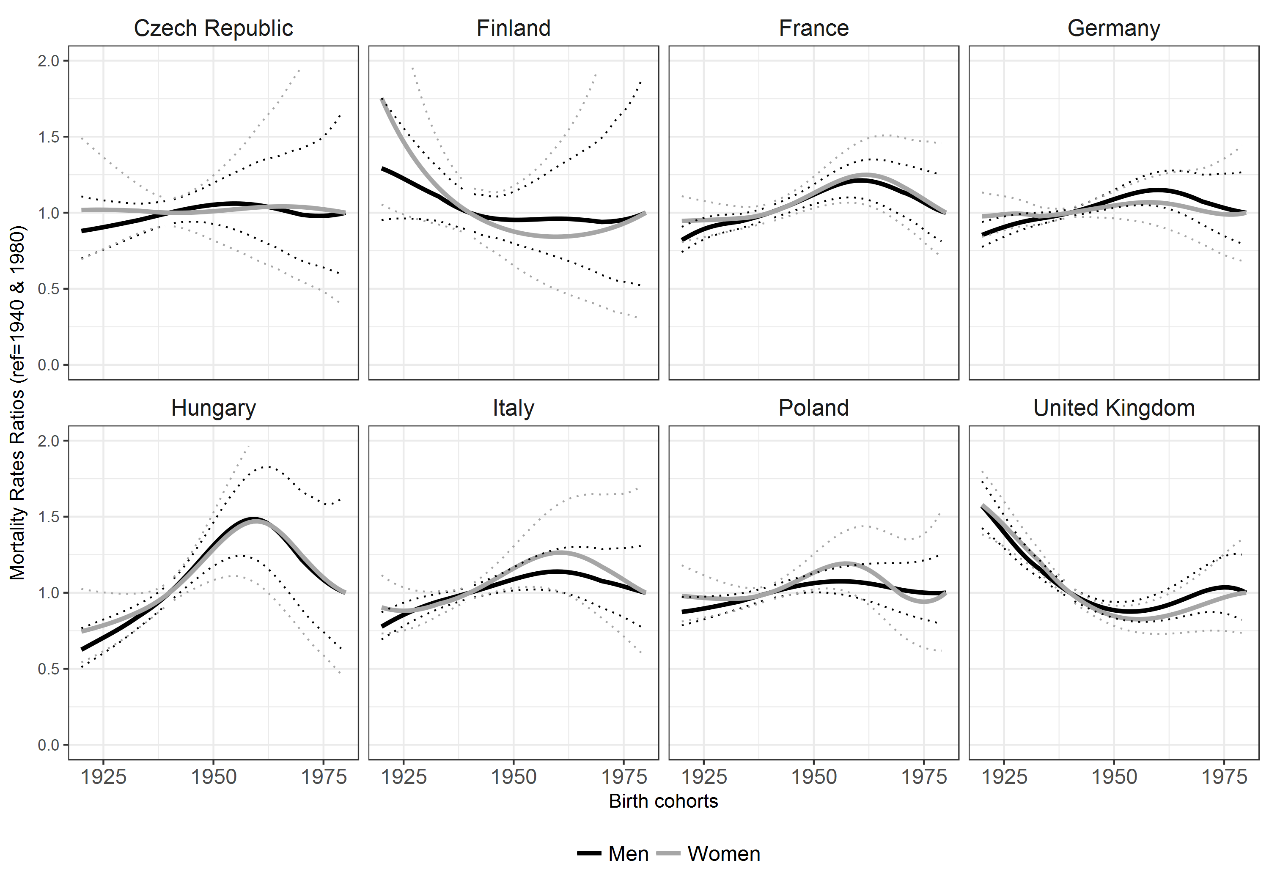

Supplement: Supplementary file 1 — Supplementary material 1 (DOCX 618 kb) [file 38_2018_1126_MOESM1_ESM.docx]
